# Neural underpinnings of morality judgment and moral aesthetic judgment

**DOI:** 10.1038/s41598-021-97782-7

**Published:** 2021-09-14

**Authors:** Qiuping Cheng, Xue Wen, Guozhen Ye, Yanchi Liu, Yilong Kong, Lei Mo

**Affiliations:** 1 School of Psychology South, China Normal University, Tianhe District, No. 55 West Zhongshan Avenue, Guangzhou, 510631 China; 2 Key Laboratory of Brain, Cognition and Education Sciences (South China Normal University), Ministry of Education, Guangzhou, China; 3 Center for Studies of Psychological Application, South China Normal University, Guangzhou, 510631 China; 4 Guangdong Key Laboratory of Mental Health and Cognitive Science, South China Normal University, Guangzhou, 510631 China; 5 School of Psychology, Hainan Normal University, Haikou, China; 6 School of Music, South China Normal University, Guangzhou, China

**Keywords:** Human behaviour, Morality

## Abstract

Morality judgment usually refers to the evaluation of moral behavior`s ability to affect others` interests and welfare, while moral aesthetic judgment often implies the appraisal of moral behavior's capability to provide aesthetic pleasure. Both are based on the behavioral understanding. To our knowledge, no study has directly compared the brain activity of these two types of judgments. The present study recorded and analyzed brain activity involved in the morality and moral aesthetic judgments to reveal whether these two types of judgments differ in their neural underpinnings. Results reveled that morality judgment activated the frontal, parietal and occipital cortex previously reported for motor representations of behavior. Evaluation of goodness and badness showed similar patterns of activation in these brain regions. In contrast, moral aesthetic judgment elicited specific activations in the frontal, parietal and temporal cortex proved to be involved in the behavioral intentions and emotions. Evaluation of beauty and ugliness showed similar patterns of activation in these brain regions. Our findings indicate that morality judgment and moral aesthetic judgment recruit different cortical networks that might decode others' behaviors at different levels. These results contribute to further understanding of the essence of the relationship between morality judgment and aesthetic judgment.

## Introduction

When you see a man rescuing the drowning child, you might be impressed by the kindness of this man's behavior. Besides that, you might also think that this man is a beautiful person on his inside. Similarly, when you see a man vandalizing flowers and trees in public, you may feel angry with this person for his bad behavior; at the same time, you may also think that this man is an ugly person on his inside. In the process of evaluating the actions in the above scenarios, people actually make two kinds of judgments on the same behavior. One kind is morality judgment, that is, people make a judgment about an individual’s social behavior as morally good/bad decision-making process^1^, which usually refers to the evaluation of the moral behavior’s ability to affect others’ interests and welfare. Goodness and badness are a very common dichotomy in ethics, philosophy, and psychology. In the general context, badness is the absence or antithesis of goodness. It is driven by fear and manifests itself through violence and division.

Another is moral aesthetic judgment, that is, the decision-making process of making a judgment about whether an individual performing good or bad behavior is intrinsically beautiful or ugly^2–6^. Moral beauty is also called spiritual beauty or inner beauty; moral ugliness is also called spiritual ugliness or inner ugliness. From a theological point of view, spiritual beauty exists whenever the attributes of God (love, justice, mercy, truth, generosity, grace) are manifested. From a philosophical point of view, human virtue is a sign of the spiritual beauty. For example, in Aristotle's Ethics, he describes human virtues as moral beauty, and the purpose of all virtues is beauty. For Aristotle, a person with good character is one who often uses spiritual beauty to guide his or her behavior, and beauty is the highest goodness in human behavior^7^. Although ugliness is a topic that has been largely neglected by aestheticists because of the intense uneasiness associated with saying ugliness, a long intellectual tradition still uses ugliness as a mark of bad character, such as the ugly stepmothers and stepsisters of Grimm's fairytales. The aesthetic judgment is emotional. According to Kant, aesthetic judgment starts from the individual object to reflect whether its form can cause some universal pleasure, in which the intermediary between the object and the subject is pleasant and unpleasant emotion^8^. Moral aesthetic judgment often implies an appraisal of the moral behavior’s capability to provide aesthetic pleasure.

Haidt and Keltner define moral beauty as "the ability to discover, recognize, and enjoy the presence of goodness in society". He says, "When we see or imagine acts of charity or gratitude, we are impressed by its beauty and we also have a strong desire to do acts of charity and gratitude"^9,10^. This strong desire is called moral elevation, of which neural mechanism was found to be in line with the neural mechanism of mentalizing^11–14^, both activate the mPFC/SFG and bilateral TPJ implicated in theory of mind (ToM), as well as the ACC, PCC/precuneus and anterior insula involved in empathic process. According to Haidt and Keltner, moral beauty is a beauty (ugliness) that people experienced in the face of virtuous (evil) behavior or the expression of moral goodness (badness) in society^15,16^. Some studies have shown that aesthetic processing usually distinctly activate the cortical network involved in the theory of mind (ToM) and empathy^6,17–19^, and better understanding of another’s intentions and emotions is related to greater aesthetic appreciation^20^.

Beauty and goodness, as well as ugliness and badness, are intrinsically linked. Traits such as honesty and kindness, or selfishness and cowardice, are imperceptible psychological traits, the goodness or badness of which stems from adherence to or violation of rational principles. If a person is morally good then, to this extent, they are beautiful; or, conversely, if a person is morally bad then, to this extent, they are ugly^21^. In recent years, more and more researchers have begun to use brain imaging technology to investigate the relationship between the neural mechanisms of morality judgment and aesthetic judgment and the brain regions. Tsukiura and Cabeza used fMRI technology to scan the responses of subjects when they judged the beauty of faces and judged the goodness of hypothetical moral behaviors, in order to investigate the neural mechanisms of aesthetic judgment and morality judgment. They found that the processing of morality and aesthetic judgments was related to the medial orbitofrontal cortex (mOFC) and insula activity^22^. The better the moral behavior described and the more beautiful the face, the stronger the mOFC activity but the weaker the insula activity; on the contrary, the worse the moral behavior described and the uglier the face, the stronger the activity in the insula but the weaker the activity in the mOFC. Wang et al. (2015) observed the relationship between the neural correlates of moral perception and facial aesthetic judgment and the brain activity in the ITG, mOFC and SFG. They suggested that two types of aesthetic judgments both involved the joint participation of perceptual, emotional and cognitive components^6^. Luo et al. asked subjects to make an integrated aesthetic judgment of facial portrait and moral description to investigate the neural underpinnings of the integration of facial beauty and moral beauty, and found that the appreciation of facial beauty and moral beauty recruited a common network involving the mOFC and middle occipital gyrus (MOG). They suggested that brain regions associated with sensory perception and reward might be recruited in the integrated aesthetic judgments^23^. Heinzelmann et al. suggested that all studies of morality judgment and aesthetic judgment involved different stimulus material and differences in visual processing of assessing the two judgment types, which make them vulnerable to confound, and as a result, any difference in neural activity between two types of judgments may be due to differences between stimulus materials, rather than the difference between morality and aesthetic judgments^24^. They measured brain activity of subjects judging the beauty of artistic images and the moral goodness of the actions depicted in the same images, and found that both common and distinct representations for morality and aesthetic judgments in temporoparietal and prefrontal regions were activated. Our recent study required subjects to rate the same positive moral behavior in terms of the good and beautiful extent to investigate the relationship between moral goodness and moral beauty from the neural perspective^17^. We found that lateral OFC was commonly involved in the processing of both goodness and beauty. Furthermore, compared with moral goodness, moral beauty specifically activated cortical network which was implicated in the processing of mentalizing. Moral aesthetics seems to be related to the internal processes that underlie a perceiver’s aesthetic experience^25^.

The above results have important enlightening significance for us to understand the relationship between morality judgment and moral aesthetic judgment through the study of neural mechanism. However, there are still some limitations in previous studies. Firstly, researchers have only studied the neural mechanism of moral process and aesthetic process respectively, but have not directly compared these two processes of morality judgment and moral aesthetic judgment under the same condition. Secondly, previous studies on the relationship between the two processes were usually local comparisons and lack of integration. For example, we have recently investigated the neural mechanisms of moral goodness and moral beauty and found that they relied on both common processes as well as distinct cognitive components. Goodness and beauty belong to one aspect of morality judgment and moral aesthetic judgment respectively, their results did not represent the neural underpinnings of morality judgment and moral aesthetic judgment. In addition to the above limitations, the research paradigms and technology used are also different, and the results of different studies differ greatly, which is difficult to integrate. Therefore, the major question whether the processes of morality judgment and moral aesthetics are the same has not been truly answered. To solve this question, the present study used the method of parameter design to record and analyze the brain activity of moral brain in the process of making morality judgment about the goodness or badness of moral behavior and the brain activity of aesthetic judgment about the beauty or ugliness of the same moral behavior, aiming to reveal the neural underpinnings of morality judgment and moral aesthetic judgment. Morality judgment will be compared with moral aesthetic judgment on the same stimulus material. Parameter design has proved valuable in exploring the relationship between experimental parameters and physiological responses to system changes^26^. Based on previous research results and theoretical discussion, we hypothesized that, on one hand, morality judgment and moral aesthetic judgment might share the common process, for example, in addition to what we've shown is the shared mechanism of moral beauty and moral goodness^17^, moral ugliness might rely on similar neural mechanisms with moral badness; on the other hand, but that compared with morality judgment, moral aesthetic judgment might involve more complex and advanced brain networks generally engaged in the process of the internal processes that underlie a perceiver’s aesthetic experience.

## Materials and methods

### Subjects

Twenty-five female college students (Mean age = 21 years, SD = 1.7) from South China Normal University were recruited to participate in this study. All subjects were right-handed, had normal vision or corrected vision, had no history of neurological or psychiatric problems. Some studies have found that women rated heterosexual faces more consistently than men^22,23^, and women are more emotional than men and more easily moved by moral events^5,27–30^. Therefore, the experimental materials designed in this study were all male moral behaviors and the recruited subjects were all female, in order to eliminate the possible confusion between the gender of the subjects and the gender of the protagonist in the experimental stimulus^23,31^. In addition, the results of our pilot experiment, which asked subjects to make subjective judgment about the degree of moral behavior, also confirmed the above findings, indicating that women's evaluations of men's moral behavior were more consistent than men's evaluations of women's moral behavior, and were able to distinguish between different degrees of moral behavior. All the subjects signed the informed consent before the start of the experiment and were paid a certain amount of money after completing the experiment. The purpose and procedure of the experiment have been approved by the Academic Committee and the Ethical Review Board of the School of Psychology, South China Normal University. All methods used in the current study were performed in accordance with the relevant guidelines and regulations of the ethical review board. The data from three subjects were excluded from analyses because of large head movements (> 3 mm maximum displacement or 3° rotation). Thus, our fMRI analyses included data from 22 subjects with an average age of 21 years (s.d. = 1.7).

### Stimuli

**Figure 1:**
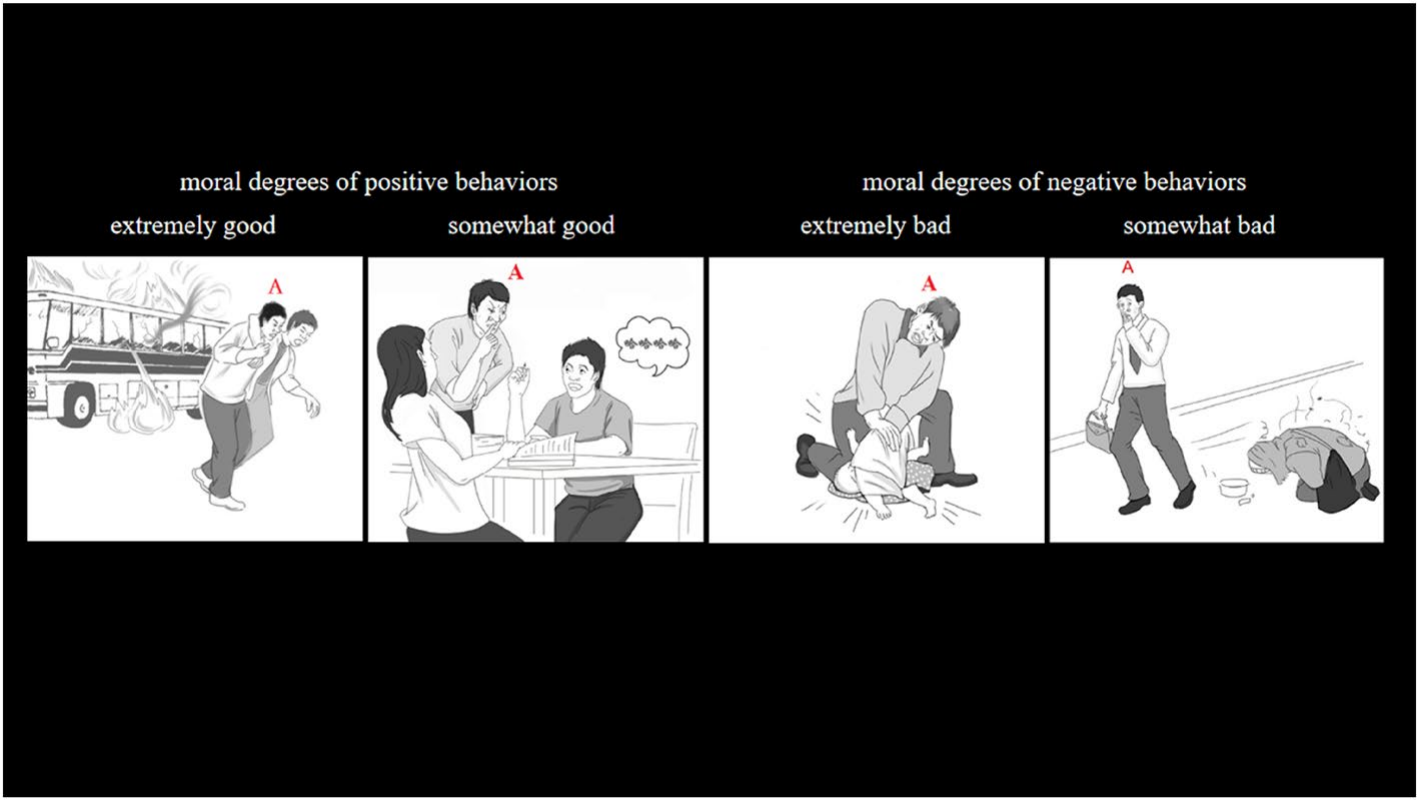
Examples of stimuli in the present experiment. The left two scenarios depict two degrees of morally positive behaviors, in which the male protagonists are performing extremely good and somewhat good behaviors, and the right two scenarios demonstrate two degrees of morally negative behaviors, in which the male protagonists are performing extremely bad and somewhat bad behaviors.

The experimental stimuli consisted of a series of scenes depicting moral behaviors of the male protagonists in the daily life. We first collected short sentences describing positive (he helped an old man cross the road, he escorted the injured to the hospital, etc.) and negative (he beat an old man, he cheated in the examination, etc.) moral behaviors. Then, scene drawings were drawn based on the sentence content. The protagonist in the scene drawing is marked with a red capital letter A to distinguish the other character(s) in the scene (See Fig. 1).

Twenty-four female college students were recruited to evaluate the effectiveness of the experimental materials. The sample size was determined with a power analysis using the G*Power tool^32^. This analysis indicated that a sample size of 24 participants would provide 80% power, reaching a minimum recommended power value^33^. The rating experiment asked subjects to rate the degree of moral behavior of the protagonist in the scene drawing on a 5-point scale (1 = extremely good, 5 = extremely bad). Based on the results, we selected 256 scenarios depicting two different moral degrees of positive behaviors (extremely good and somewhat good) and 256 scenarios depicting two different moral degrees of negative behaviors (extremely bad and somewhat bad), each containing 128 scenarios. A total of 512 scene drawings were used as stimulus materials for the following fMRI experiment. The scene drawing was 589 × 500 pixels.

Scenarios of positive and negative moral behaviors were pseudorandomly divided into two groups respectively. Half of positive behavior and half of negative behavior scenes were used as stimulus material for morality judgments, and another half of positive behavior and another half of negative behavior scene were used as stimulus material for moral aesthetic judgments, in order to avoid using the same stimulus material for different judgment tasks. The order of the two groups was counterbalanced across subjects. Each group contains 128 scenarios. The data from two subjects were excluded from analyses because of two much missing values. A 2 (group 1 and group 2) × 2 (behavior nature: positive behavior and negative behavior) × 2 (moral degree: extremely and somewhat) repeated measure ANOVA was used to test whether the two groups of material were equivalent. Evaluation results show that the main effect of group was not significant, F (1, 21) = 0.008, p > 0.05, the main effect of behavior nature was significant, F (1, 21) = 1202.34, p < 0.001, η^2^_p_ = 0.98, the main effect of moral degree was significant, F (1, 21) = 85.54, p < 0.001, η^2^_p_ = 0.8. The interaction between behavior nature and moral degree was significant, F (1, 21) = 1265.01, p < 0.001, η^2^_p_ = 0.98, which was driven by the significant difference of the moral degrees of positive moral behaviors, t (21) = 17.85, ps < 0.001, and also the significant difference of the moral degrees of negative moral behaviors, t (21) = 31.24, ps < 0.001. The two-way interactions between behavior nature and group (F (1, 21) = 0.058, p > 0.05), between group and moral degree (F (1, 21) = 0.003, p > 0.05), and the three-way interaction between group, behavior nature and moral degree (F (1, 21) = 1.19, p > 0.05) were all not significant. This result indicates that positive and negative moral behaviors can be distinguished well, and there is no significant difference between the two groups of materials, which ensures the equivalence of the two groups of materials. This result is important because it suggests that differences in brain activity involved in morality processing and moral aesthetic processing are not caused by differences in experimental stimuli.

Therefore, each judgment task contains scenarios of positive and negative behaviors with two different moral degrees. The allocation of these materials is balanced among the different judgment tasks. The moral degrees of the behaviors involved in each task were used as a parameter to determine the brain activation changes that varied with the degree of morality in the judgment tasks.

### Procedure

The experiment consists of two tasks: morality judgment (MJ) and moral aesthetic judgment (MA). In the morality judgment, the subjects were asked to rate the degree of goodness or badness of the moral behavior in the scenarios on a 4-point scale (1 = extremely good, 4 = extremely bad); In the moral aesthetic judgment, the subjects were asked to rate the degree of beauty or ugliness of the moral behavior in the scenarios on a 4-point scale (1 = extremely beautiful, 4 = extremely ugly), while their brain activity was scanned. Scan was an event-related design.

**Figure 2:**
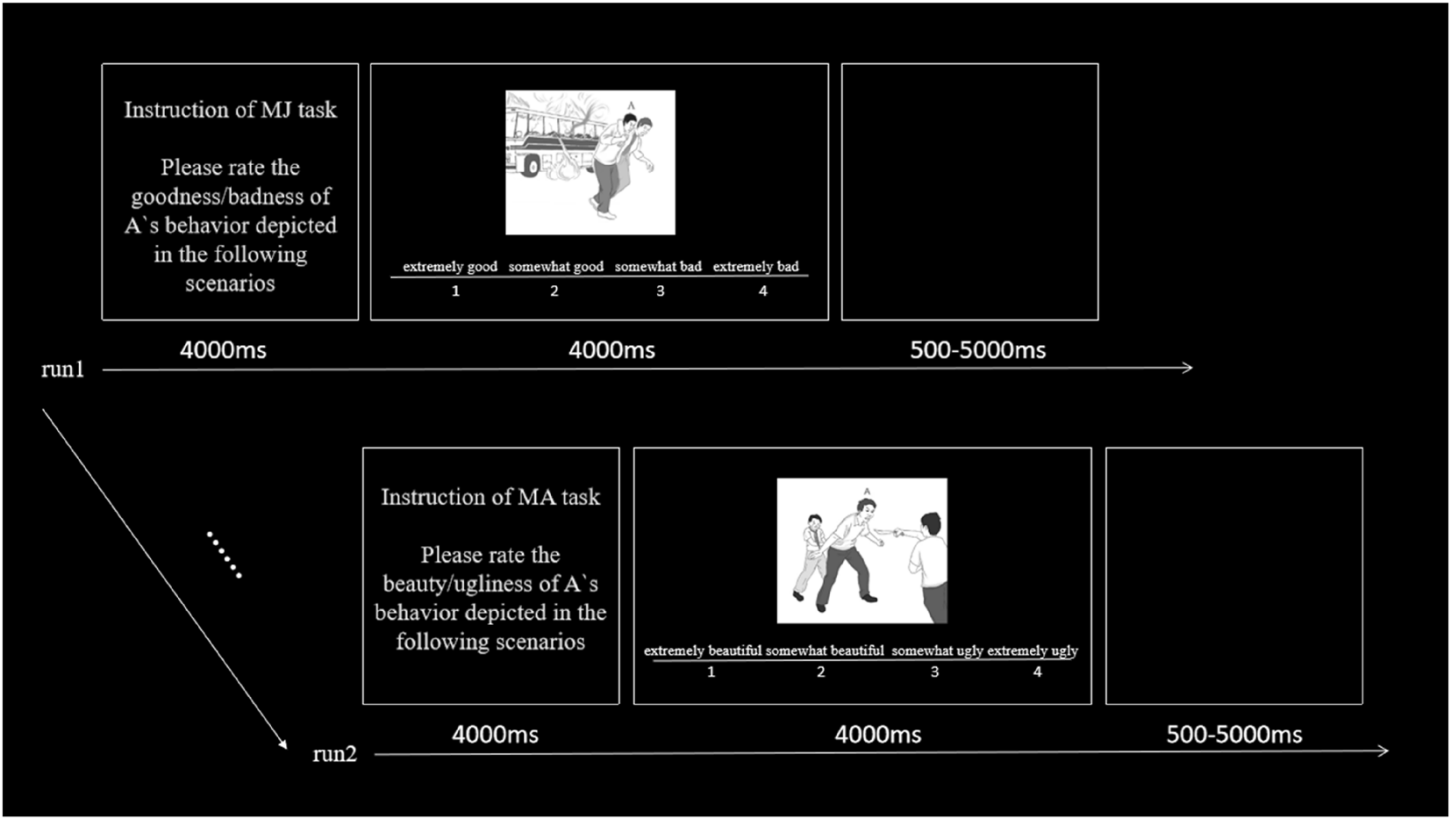
The experimental flowchart. Stimuli were scene drawings of positive or negative behaviors with two different moral degrees. Subjects were required to rate the degree of goodness or badness about the morally positive behaviors or negative behaviors in the morality judgment, as well as to rate the degree of beauty or ugliness about the morally positive behaviors or negative behaviors in the moral aesthetic judgment. The order of the two tasks was counterbalanced across subjects.

During the MRI scan, the task instructions of 4 s were presented at the center of the computer screen, which told the subjects about the upcoming task. Next, one scenario was presented with a 4-point scale under it in which the number indicated the corresponding moral degree. The subjects were instructed to assess the moral degree of behavior of the protagonist (red A marked) in the scene drawing as quickly as possible within 4000 ms. Using a response box with buttons labeled 1–4, half of the subjects made a left index finger response (i.e., 1 or 2) for positive behaviors and a right index finger response (i.e., 3 or 4) for negative behaviors. Reverse responses were used for the other half of subjects to balance any influence of movement on data. Once the response was recorded, the rating bar disappeared and was followed by a blank display for a random period ranging between 500 and 5000 ms. If the scenario slide was not presented for 4000 ms after subjects pressed the button, it disappeared and a blank display will appear for the remaining time (See Fig. 2). The maximum duration of the presentation of the scene drawing slide was 4000 ms, based on the results of a pilot test. Each run was scanned for approximately 16 min. Each task had two runs, with 128 trials per run. Each run consisted of four conditions, each of which had 32 trials. The whole experiment took about 70 min to run.

### fMRI data acquisition

A Siemens 3 T Trio Scanner with a 12-channel head coil at the Brain Imaging Center of South China Normal University was used to collect the MRI image data, and a special pad was used to fix the head and prevent the head from moving. The T2* weighted functional images were obtained by using the echo plane imaging sequence. The specific scanning parameters were as follows: 33 layers of the whole brain image were scanned, each layer thickness was 3.5 mm, layer spacing was 0.8 mm, TR = 2000 ms, TE = 30 ms, Flip Angle = 90°, FOV = 204 × 204 mm^2^, Acquisition matrix = 64 × 64. High resolution T1-weighted structural images were obtained by using the MP-RAGE sequence, with specific parameters as follows: TR = 1900 ms, TE = 2.52 ms, Flip Angle = 9°, FOV = 256 × 256mm^2^, Acquisition matrix = 256 × 256, thickness = 1.0 mm, number of layers = 176, voxel size = 1 × 1 × 1 mm^3^. All subjects were asked to keep their heads still and stay awake during the MRI scan.

### fMRI data analysis

Data preprocessing and statistical analyses of all images were conducted using SPM12 (Welcome Trust Center for Imaging, London, UK; http://www.fil.ion.ucl.ac.uk/spm). For each run of each participant, the preprocessing analysis consists of the following steps: (1) interscan slice-time correction, (2) spatial realignment and motion correction, (3) spatial normalization of aligned functional images to the Montreal Neurological Institute (MNI) template and resampling with a voxel size of 2 × 2 × 2 mm^3^, and (4) spatial smoothing with an isotropic Gaussian kernel of 6 mm FWHM. The three-dimensional translation of all the subjects' heads was less than 3.0 mm, and the three-dimensional rotation was less than 3.0°.

Statistical analysis was performed after preprocessing. We used parametric design^26^ to study the BOLD response pattern of the whole brain to MJ and MA judgments. In this study, we manipulated the moral degree of the protagonist's behavior in the scene drawing to explore the neural underpinnings related to the processing of morality judgment and moral aesthetic judgment.

At the individual level, the trial-related activity observed in each subject was modeled by convolving a vector of trial onsets with a canonical hemodynamic response function (HRF) within the context of the General Linear Model (GLM). We input the rating scores for moral degree of each behavior into the SPM12 parametric modulator to identify brain regions displaying altered activity simply as a function of MJ and MA ratings. The missed trials were not modeled as predictors. On a given trial, the duration of the predictor of interest is equal to 4 s as shown in the scenario. Six head movement parameters calculated during the realignment were added to the model as non-interest predictors to offset the effects of head movement. Trials that did not respond to were not added to the model and were therefore included in the baseline. Low frequency noise was removed by 1/128 Hz high pass filter (HPF). For each subject, the brain regions associated with the changes in the degree of morality were identified by comparing the parameter-adjusted predictors of interest to baseline.

In the first-order analysis, each subject was analyzed separately. Then the first-level individual contrast images were fed to the second-level analysis employing a whole-brain random-effects model. Firstly, the cortical networks processed by MJ and MA were determined by parameter analysis, separately. Secondly, we calculated the conjunction analysis of MJ and MA contrasts to identify the common brain regions involved in the processing of two types of judgments. Third, we calculated a direct comparison between MJ and MA to obtain their unique brain activity. In addition, the parameter estimates in the regions of interest (ROI) was obtained through leave-one-subject-out-cross-validation (LOOCV) analysis. In this analysis, in each iteration, the ROI of the excluded subjects was defined using data from all subjects except one, which served as the test set^6,34,35^. Each ROI was defined using a sphere with 6 mm radius centered on the peak voxel. This procedure was repeated 22 times by omitting a different subject each time, and the parameter estimates were then averaged across subjects and plotted. The LOOCV analysis method shows that the data used to define the ROI and the data extracted from that ROI are independent. We report the neural results at a voxel-level threshold of p < 0.05 (FDR-corrected) and cluster-level threshold of p < 0.05 (FWE-corrected) to correct for multiple comparisons. Statistical maps were labeled based on the MRIcro atlas (http://www.mricro.com), and results were visualized with the BrainNetViewer (http://www.nitrc.org/projects/bnv/)^36^.

## Results

### Behavior results

The mean rating scores and the mean response times (RTs) of ratings for each moral degree in MJ and MA tasks were calculated and subjected to two 2 (task: MJ and MA) × 4 (moral degree: (1) extremely good/beautiful, (2) somewhat good/beautiful, (3) somewhat bad/ugly, and (4) extremely bad/ugly) repeated measures ANOVAs. In this analysis, half of the subjects' ratings were converted to the same criteria as the other half (i.e. reverse coding), with a larger number representing particularly bad or particularly ugly. Greenhouse–Geisser correction was performed on the non-sphericity of the data as needed, and the uncorrected degrees of freedom, the corrected p value, and the effect size (η^2^_p_) were reported. Bonferroni correction was used to adjust the p-value of the paired comparison. In all analyses, the significance level was set at 0.5. The results of rating scores showed that the main effect of task was significant, F (1, 21) = 6.2, p = 0.02, η^2^_p_ = 0.23, with significantly higher score of MJ than that of MA. The main effect of moral degree was significant, F (1, 21) = 274.14, p < 0.001, η^2^_p_ = 0.93, with significant differences between degree 1 and degree 2, t (21) = 17.62, p < 0.001, as well as degree 3 and degree 4, t (21) = 22.63, p < 0.001. The interaction was not significant, F (1, 21) = 0.029, p > 0.05. The results of the response times (RTs) of ratings showed that the main effect of task was not significant, F (1, 21) = 0.13, p > 0.05. The main effect of moral degree was significant, F (3, 63) = 13.82, p < 0.001, η^2^_p_ = 0.397, with the stronger the moral degree, the faster the response. The interaction was not significant, F (1, 21) = 0.058, p > 0.05. This result is important because it shows that the fMRI analysis focused on the activity of the brain regions involved in the processing of MJ and MA and was not confused by differences in task difficulty. This result is also critical because it avoids the correlation in GLM and independently explains the effect of the two processes on the BOLD signal strength.

### Imaging results

**Figure 3:**
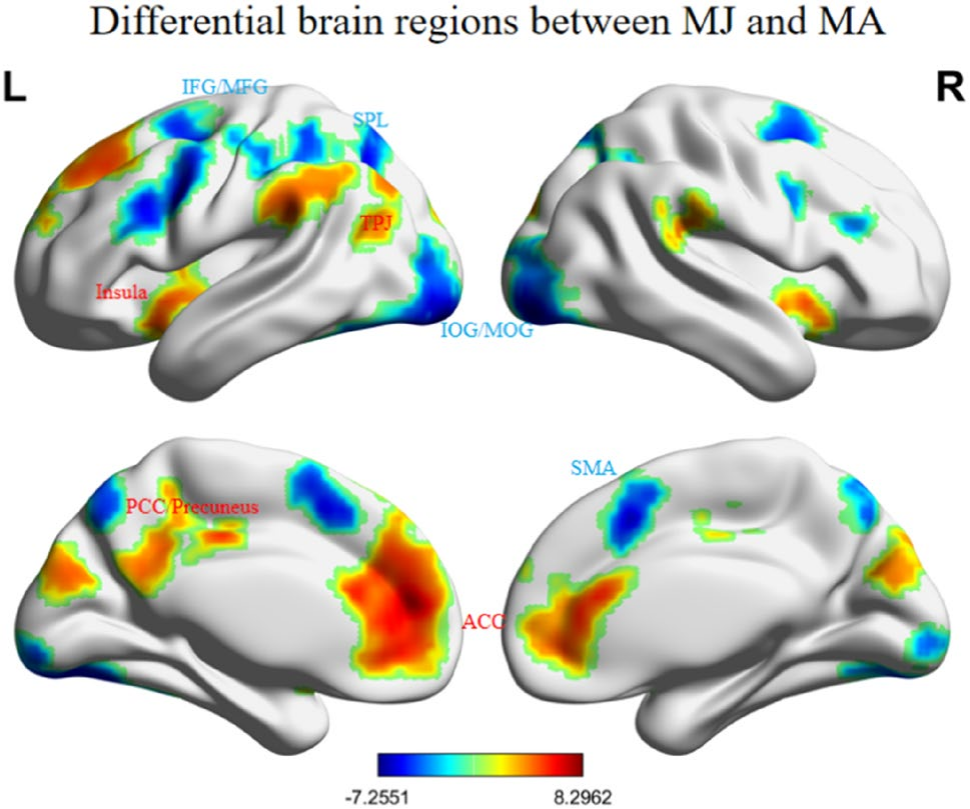
Differences between MJ and MA at a threshold of p < 0.05 (FWE-corrected). Images were plotted with the BrainNet Viewer. Warm (Cold) color indicates that MA elicited greater (weaker) activity than MJ.

According to the purpose of this experiment, we used parameter design to determine the neural activity of MJ and MA by inputting the score data for moral degree of each behavior obtained from morality judgment and moral aesthetic judgment into the SPM12 parametric modulator. The MJ that varies with the degree of good and bad significantly elicited activation within the Precentral Gyrus (ventral premotor Cortex, PMv), Supplementary Motor Area (SMA), right Inferior/Middle Occipital Gyrus (IOG/MOG), right Inferior Parietal Lobule (IPL), right fusiform gyrus probably corresponding to the so-called fusiform face area (FFA)^37^, and right Inferior Frontal Gyrus (IFG), extending onto the crossing between lateral Orbitofrontal Cortex (lOFC) and insula. The MA that changes with the degree of inner beauty and ugliness significantly elicited activation the Anterior Cingulate Cortex/Medial Prefrontal Cortex/Medial Orbitofrontal Cortex (ACC/mPFC/mOFC), Posterior Cingulate Cortex/precuneus (PCC/Precuneus), bilateral insula and Temporo-Parietal Junction (TPJ). Conjunction analysis of the parametric modulated MJ and MA revealed no significant brain region overlap. The direct contrast revealed that a number of areas were differentially activated by the two categories of judgments tasks under investigation (See Fig. 3 and Table 1). Morality judgment (MJ-MA) elicited activation within the bilateral IOG/MOG, MFG/IFG extending into the PMv, SMA and Superior Parietal Lobule (SPL). In contrast, moral aesthetic judgment (MA-MJ) caused bilateral activation within insula, ACC/mPFC/mOFC, PCC/Precuneus, Middle Temporal Gyrus (MTG)/Angular and TPJ.

**Table 1:** Coordinates, voxel sizes and peak values showed the activated brain regions of MJ and MA (p < 0.05, FWE-corrected).

Regions	R/L	BA	x	y	z	*t* score	size
**The Comparison of Morality Judgment (MJ) and moral aesthetic judgment (MA)**							
Parametric_modulated_MJ							
Precentral Gyrus/PMv	R/L	6	−39	−9	66	6.11	969
IFG/lOFC/Insula	R	9/46/13	33	24	3	5.78	850
SMA	R/L	8	6	18	45	5.64	256
MFG	R	10	33	54	6	4.32	125
IOG/MOG	R	18/19	33	−87	−12	4.71	95
IPL	R	4/6	30	−54	45	4.44	60
FFA	R	37	39	−45	−18	4.19	52
Parametric_modulated_MA							
ACC/mPFC/mOFC	R/L	32	6	39	36	6.17	904
Cuneus/precuneus	R/L	31	12	−75	24	4.59	196
TPJ	L	40	−57	−51	36	5.79	158
Insula/IFG	R	47/13	30	21	−9	5.37	126
Insula/IFG	L	13/47	−30	15	−15	5.64	104
TPJ	R	40	60	−42	30	4.04	89
Conjunction of MJ ∩ MA							
No activation							
**Differences between MJ and MA**							
Specific regions of MJ_[MJ > MA]							
MFG/IFG/PMv	L	6/9	−48	3	33	6.87	693
IOG/MOG	L	18	−27	−93	−12	7.25	613
SPL/IPL	L	7	−21	−63	45	6.69	592
IOG/MOG	R	18	33	−87	−12	7.1	480
SMA	R	32	6	18	45	6.41	268
SPL/IPL	R	7	27	−57	45	4.91	132
MFG/IFG/PMv	R	9/8	54	15	33	4.5	96
Specific regions of MA_[MA > MJ]							
ACC/mPFC/mOFC	L/R	32/9/8	−6	45	12	8.3	1419
PCC/precuneus	R/L	31	−6	−24	39	4.59	670
TPJ	L	40/39	−54	−39	30	6.02	276
TPJ	R	40	66	−36	18	4.88	186
Insula	L	13	−42	6	−6	6.13	183
MTG/Angular	L	39	−39	−69	21	4.63	176
Insula/IFG	R	47/13	39	12	−6	5.78	100

Note: The t-scores computed by SPM12 quantify the statistical difference between the two conditions. Coordinates refer to the stereotactic space of the Montreal Neurological Institute. MFG Middle Frontal Gyrus, IFG Inferior Frontal Gyrus, SMA Supplementary Motor Area, IOG/MOG Inferior/Middle Occipital Gyrus, SPL/IPL Superior/Inferior Parietal Lobule, FFA fusiform face area, ACC/PCC Anterior and Posterior Cingulate Cortex, mPFC Medial Prefrontal Cortex, mOFC medial Orbitofrontal Cortex, TPJ Temporo-Parietal Junction, MTG Middle Temporal Gyrus.

### ROIs analysis

**Figure 4:**
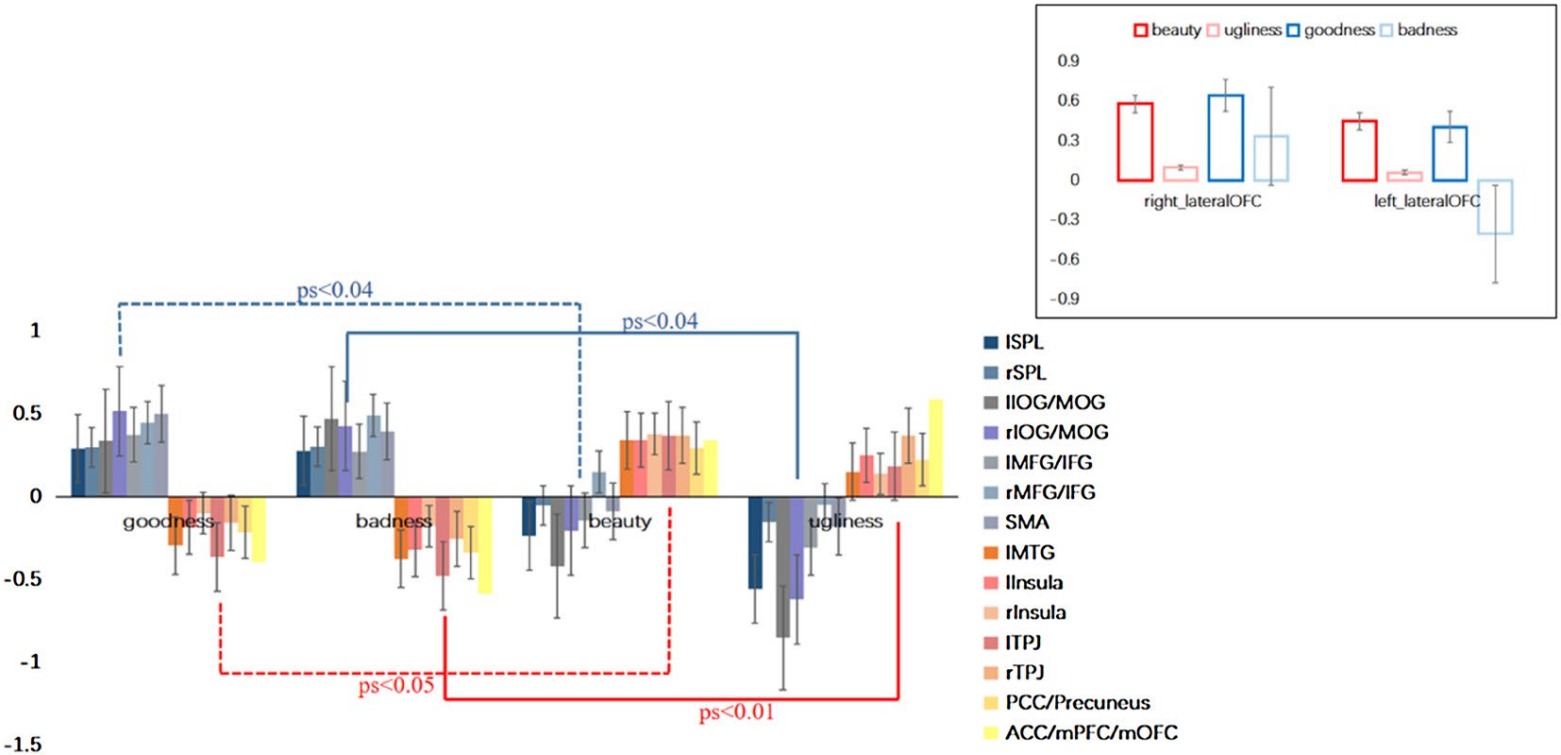
ROIs results of brain regions involved in the processes of MJ and MA that respond to goodness, badness, beauty and ugliness. The parameter estimates (β values) were extracted from the defined ROIs of bilateral SPL, bilateral IOG, bilateral MFG/IFG and SMA (cold color), as well as the ACC/mPFC/mOFC, PCC/precuneus, bilateral TPJ and bilateral insula (warm color), and the bilateral lateral OFC (the upper left corner), then one-way repeated measure ANOVAs were performed. Error bars indicate standard error of means.

In order to further investigate whether the above significant activated clusters selectively respond to the four categories of goodness, badness, beauty and ugliness during the process of MJ and MA, we performed a LOOCV analysis. ROIs were defined as a 6 mm sphere centered on the peak voxel in areas identified by direct task contrasts MJ-MA and MA-MJ. Our recent study on the neural mechanisms of moral goodness and moral beauty^17^ found the common involvement of left lateral OFC (MNI peak coordinates: − 36, 24, − 15) in the processing of moral goodness and moral beauty. In the present study, we found that both morality judgment and moral aesthetic judgment activated the right lateral OFC (MNI peak coordinates: 30, 27, 3), although the result of the conjunction analysis showed that there was no shared brain region between these two types of judgments. It is necessary to further investigate the response pattern of bilateral lateral OFC in the four categories of goodness, badness, beauty and ugliness. Therefore, ROI analysis was also conducted on these two brain regions. Each ROI was iteratively defined on n-1 subjects, and then β value (parameter estimates) of excluded subjects were extracted from above defined ROIs. One-way repeated measures of ANOVA showed that there were significant differences in four categories of reactions on the left MFG/IFG (F(3,72) = 8.34, p < 0.001, η^2^_p_ = 0.26), left SPL (F(3, 72) = 8.56, p < 0.001, η^2^_p_ = 0.26), bilateral IOG (lIOG, F(3, 72) = 12, p < 0.001, η^2^_p_ = 0.33; rIOG, F(3, 72) = 10.21, p < 0.001, η^2^_p_ = 0.3) and SMA (F(3, 72) = 6.67, p < 0.001, η^2^_p_ = 0.22), which were mainly driven by a higher signal for good response as compared to beautiful response, t(21) < 3.23, ps < 0.04, a higher signal for bad response as compared to ugly response, t(21) < 5.16, ps < 0.04. And the BOLD signal changes between goodness and badness in these ROIs were indifferent, ps > 0.05. There were also significant differences in four categories of reactions on the left insula (F(3, 72) = 6.99, p < 0.001, η^2^_p_ = 0.23), bilateral TPJ (lTPJ, F(3, 72) = 9.63, p < 0.001, η^2^_p_ = 0.29; rTPJ, F(3, 72) = 5.81, p < 0.001, η^2^_p_ = 0.2), ACC/mPFC/mOFC (F(3, 72) = 17.71, p < 0.001, η^2^_p_ = 0.43) and PCC/precuneus (F(3, 72) = 10.15, p < 0.001, η^2^_p_ = 0.3), however, which were mainly driven by a higher signal for beautiful response as compared to good response, t(21) < 3.91, ps < 0.05, a higher signal for ugly response as compared to bad response, t(21) < 6.18, ps < 0.01. And the difference between beauty and ugly was not significant (See Fig. 4). These results demonstrated that the brain regions involved in the process of MJ were only sensitive to good and bad responses, while the brain regions involved in the process of MA were only sensitive to beautiful and ugly responses.

In addition, differences in the four response categories were also significant in the bilateral lateral OFC (See an Image inserted in the upper left corner of Fig. 4) (lOFC, F(3, 72) = 16.82, p < 0.001, η^2^_p_ = 0.29; rOFC, F(3, 72) = 5.21, p = 0.002, η^2^_p_ = 0.11). In the left lateral OFC, the differences were mainly driven by a higher signal for good response as compared to bad response, t(21) = 6, p < 0.001, a higher signal for beautiful responses as compared to ugly, t(21) = 2.75, p = 0.04, and bad responses, t(21) = 6.23, p < 0.001; in the right lateral OFC, the differences were mainly driven by a higher signal for good response as compared to bad response, t(21) = 2.13, p = 0.04, a higher signal for beauty response as compared to ugly response, t(21) = 2.73, p = 0.009. These results revealed that the bilateral lateral OFC had stronger activation for goodness and beauty than badness and ugliness, which was consistent with a recent finding on the neural mechanism of moral goodness and moral beauty judgments^17^, namely, the lateral OFC is commonly engaged in the processing of both moral goodness and moral beauty.

## Discussion

In the present fMRI study, we required the subjects to assess the degree of goodness or badness, as well as the degree of beauty or ugliness of moral behaviors in daily life, to determine whether morality judgment and moral aesthetic judgment differ in their neural underpinnings or not. Behavioral results revealed that the rating of morality judgment was significantly higher than those of moral aesthetic judgment for the same moral behavior, indicating that people may be more inclined to make morality judgment. The fMRI results revealed that when the same experimental material was used but varying task demands were systematically considered, the distinct nervous systems of morality judgment and moral aesthetic judgment emerge with more clarity. Specifically, direct contrasts showed specific activations for morality judgment; these were mainly located in the frontal, parietal and occipital cortex, including the bilateral SMA, IPL/SPL, MFG/pars opercularis of IFG extending onto the ventral premotor cortex (PMv), and IOG/MOG: i.e., brain regions which have been previously reported for motor representations of behavior during understanding others` behaviors^38,39^. These areas are only sensitive to goodness and badness, but not to beauty and ugliness. In contrast, moral aesthetic judgment significantly elicited specific activations in the frontal, parietal and temporal cortex, including the bilateral ACC/mPFC/mOFC, PCC/Precuneus, TPJ and anterior insula: i.e., brain regions which have been proved to be involved in understanding other people's behavioral intentions and emotions^39^. These areas are only sensitive to beauty and ugliness, but not to goodness and badness. Interestingly and more importantly, when subjects judged a behavior to be good (as in contrast to bad) or to be beauty (as in contrast to ugly), the lateral OFC showed enhanced BOLD signals, which was in line with what was found in a recent study that lateral OFC was commonly involved in the processing of goodness and beauty^17^. But no common brain region was found to be involved in the processing of badness and ugliness. Against the background of the literature, present findings indicate that morality judgment and moral aesthetic judgment seemly activate completely different cortical networks, although lateral OFC is commonly activated in both moral goodness and beauty.

The OFC is critical for goal-directed behavior^40–42^. Converging neuroimaging evidence shows differences between lateral OFC and medial OFC^43–51^, the medial OFC in general is concerned with monitoring reward values^31,52–58^, but the lateral OFC connecting the anterior insula cortex is generally involved in the processing of affectively stimuli^59,60^. We found that both moral goodness and moral beauty more strongly activated the lateral OFC/anterior insula, suggesting that this brain region may be involved in encoding the emotional experiences that often occur in human interactions. As moral goodness and moral beauty judgments share a very critical characteristic, that is, the object of both judgments generally refers to the positive moral behavior. This result is not surprising given that positive moral behavior is a rewarding stimulus that evokes emotional experiences.

It is important to mention, we found that the medial OFC, with strong connections with vmPFC and ACC, not only responded to moral beauty, but also responded to moral ugliness, and there was indifferent in response intensity between them. Due to strong connections among OFC, vmPFC and ACC a network was suggested—the orbital medial prefrontal cortex (OMPFC)—related to emotion processing and social cognition^61,62^. Traditionally, the mOFC has commonly been suggested as an important reward-related region involved in the processing of beauty or positive stimuli^49,54,55,63–65^, however, recent works showed that the mOFC was activated by negative experiences^66,67^, and also was involved alike by both the beautiful and the ugly stimuli^18^. The mOFC, as a part of the medial cortical structures involved in self-referential and self-related processes^68^, has been proved not only to be involved in self-perception in social cognition^69^, but also engaged in coding internal motivational values, particularly in the absence of external prompts^70^. Consistent with the above findings, the result that both moral beauty and moral ugliness significantly activated the OMPFC might be interpreted as the activation of neural circuits related to self vs. other-assessment^18^. In the moral aesthetic judgment, people usually evaluate the inner beauty or ugliness of others through morally behavioral understanding, in which their mental status will be perceived and be compared with ourselves. And the mOFC or OMPFC might play a crucial role in this process.

In the present study, people usually focus on the action itself^71,72^ and are emotionally unmoved^2,15,16^ when they judge whether a moral behavior is good or bad. Behaviors directed by morality judgment are cognitively experienced and judged without deep emotional involvement^73^. However, when people evaluate a person's inner beauty or ugliness according to his moral behavior, people's emotions are easily affected by his behavior: that is, if he acts good, people`s emotions are elevated^5,9,10,74–76^; If he acts evil, people feel contempted, angry or disgusted^77^, and the emotion is unpleasant. Both morality judgment and moral aesthetic judgment are accomplished by behavioral understanding. Understanding the behaviors of others will contribute to the harmony of people`s interpersonal relationships, and is necessary for efficient communication and collaboration, which deals primarily with what they are doing, how they are doing it (i.e., behavioral states), and why they are doing it (i.e., mental states)^78,79^, and it is accomplished by two processes and related brain networks: motor representation of behavior and intention representation of behavior, supported by the mirror neuron system (MNS) and the mentalizing system (MZS), respectively^39^. Prior neuroimaging studies have shown that the MNS and the MZS are differentially activated by what/how and why questions about behaviors^79^.

Cortical areas with motor properties, including inferior frontal gyrus (IFG), ventral premotor cortex (PMv) and inferior parietal lobule (IPL), namely, the MNS, have been observed to respond to motor behaviors when they are performed and observed^80–88^, reflecting motor representation of behavior^89–93^. Li et al. recently used fNIRS to examine the brain activity in the frontal, motor, parietal and occipital regions, aiming to better understand the brain correlates involved in encoding motor complexity. They found that motor complexity sensitive brain regions were present in the pars opercularis IFG/PMv, primary motor cortex (M1), IPL/supramarginal gyrus and middle occipital gyrus (MOG) during behavior execution, and in pars opercularis IFG/PMv and M1 during behavior observation, suggesting that the processing of motor complexity involves not only M1 but also pars opercularis IFG, PMv and IPL, each of which plays a critical role in behavior perception and execution^84^. Some studies that used transcranial magnetic stimulation^94^ found that the left IPL is involved in the representation of the manipulability of the objects, while the temporal cortex includes more abstract representation of object function. Spun et al. found that there existed a dissociation between the posterior and anterior regions of IFG in their contribution to behavior representation^92,95,96^: The pIFG/PMv is associated with the perception of behaviors and the execution of understanding behaviors, mainly manifesting in encoding more concrete representations of behaviors—“what” and “how”; and the anterior IFG region located in the ventrolateral PFC is associated with the motive of understanding behaviors, mainly manifesting in encoding more abstract representations of behaviors—“why”. In the present study, we found that compared with moral aesthetic judgment, morality judgment, both good reaction and bad reaction, recruited motor-related areas, including MFG/IFG/PMv, SMA, SPL/IPL and IOG/MOG, indicating that morality judgments might use motor representation through “body” reading, supported by the MNS^93,97–99^ which is part of a larger sensorimotor brain network, to complete assessments of whether a moral behavior is good or bad and how good or bad it is. It is worth noting that the brain network for morality judgment is more consistent with that for the behavior execution than with that for the behavior observation.

Although the human MNS is reliably active during behavior understanding, several studies have shown that it is insensitive to the intentional representation of observed behavior; rather, a separate brain system known as the ToM or mentalizing system (MZS), by inferring the internal states of other persons that typically drive behaviors, such as their goals, desires, beliefs, intentions, causal attributions and traits, appears to be critical^90,100–102^. It is part of a larger default brain network, including the medial PFC, PCC/precuneus and the TPJ. We found no activation of brain regions associated with mentalizing in morality judgment, which is consistent with the result from a recent study^103^. In this study, Yoder and Decety scanned 40 healthy adults using fMRI while these subjects were watching scenes in which people harmed or helped others. They found that subjects sensitive to righteous actions activated brain regions associated with (social) cognitive aspects of morality judgment, rather than with processing emotional aspects of a moral scenario. Mentalizing, in particular, empathy, may not be necessary for deciding whether moral behaviors are right or wrong, but it may still play an important role in the motivation of moral behavior^104^. Morality judgment, assessing of whether a moral behavior is good or bad and how good or bad it is, seems to rely on more primitive cognitive systems^105^.

The MZS is usually activated by why questions about behaviors is responsive to tasks of mental-state reasoning^106–111^. The brain regions of the MZS are involved in the multifunctional process of distinguishing oneself from the thoughts of others and gaining an understanding of their mental status, which in turn enable people to recognize, interpret, and predict behavior^112,113^. In the present study, when the subjects were required to evaluate the beautiful or ugly degree of the moral behaviors, brain regions consistent with the MZS were activated, suggesting that the processing of moral aesthetics is likely to be supported by the MZS. In the process of moral aesthetic judgment, people are likely to understand the social actor`s moral behavior and make a judgment of beauty or ugliness by reasoning the mental status that typically drive behavior, such as beliefs, desire and intentions. Moral aesthetic judgment may require psychological reflection or, more generally, a shift in attention to the social actor's intention. The neural mechanism for the question of how beautiful or ugly the moral behavior is, seems to be the same as the neural mechanism for the question of why the behavior is done. By manipulating either behavior goals or the content of a perceived behavior^100–102^, Thioux et al. found that the MNS supported the understanding behaviors at low (how) and intermediate (what) levels of abstraction, whereas the MZS supported the understanding behaviors at high (what) levels of abstraction^114,115^.

In addition, in moral aesthetic judgment, the beauty or ugliness of moral behavior generally refers to the perception of the beautiful or ugly of social actor's personality traits^3,116^, and the traits inference always involves the activiations of TPJ and mPFC^117,118^. In addition to regions implicated in mentalizing, we found that moral aesthetics also recruited the activation of anterior insula. This region is not only implicated in the meta-representation of emotional states and interoceptive awareness^119–122^, but also correlates with the cognitive processing of self and others^123^, particularly is critical for the perceived intentionality of other people`s behavior^124^. In short, moral aesthetic judgment refers to the perceiver's judgment on the inner beauty of others, which seems to be concerned with the internal processes^125^ that underlie a perceiver’s aesthetic experience. This process has been reported to be supported by the cortical network implicated in the mentalizing system.

In brief, previous studies mainly focused on the neural mechanism of moral process and aesthetic process respectively; the present study directly compared the two processes of morality judgment and moral aesthetic judgment under the same condition and examined the commonalities and differences in the neural correlates of these two processes through an experimental approach. However, the present study is limited in that it only used right and wrong behaviors as representatives of morality judgment; future studies using different kinds of moral judgments, such as wrongness, punishment, blame, moral dilemmas, etc., should be performed to verify our findings. In addition, to eliminate the possible confusion between the gender of the subjects and the gender of the protagonist in the experimental stimulus, we used male moral behaviors as experimental materials and recruited female subjects to evaluate behaviors. It would be interesting to determine whether the same results could be found if female moral behaviors were used as experimental materials and men were recruited as subjects. Or, whether the results obtained by recruiting subjects of different races to make moral or moral aesthetic judgments would hold up cross-culturally is also very worthy to be investigated.

## Conclusions

The perception of moral aesthetics often implies an appraisal of the moral behavior’s capability to provide aesthetic pleasure, which seems to be related to the internal processes that underlie perceivers`aesthetic experience; while morality judgment usually refers to the evaluation of the moral behavior`s ability to affect the interests and well-beings of others, which seems to be related to the execution of the behavior. Both types of judgments are accomplished by behavioral understanding. In the present study, we reveal the neural underpinnings of morality judgment and moral aesthetic judgment by collecting data about subjects` brain activity as they evaluate the extent of goodness and badness, as well as the extent of beauty and ugliness of moral behaviors. By comparing two types of judgments made by the same subject on the same stimulus material, we found that morality judgment and moral aesthetic judgment recruit different cortical networks that might decode others' moral behaviors at different levels, although lateral OFC is commonly involved in the processing of goodness and beauty. These results contribute to further understanding of the essence of the relationship between morality judgment and aesthetic judgment.

## Data Availability

All data analyzed in this study are available from the corresponding author upon reasonable request.
